# Long-Lasting Rituximab-Induced Reduction of Specific—But Not Total—IgG4 in MuSK-Positive Myasthenia Gravis

**DOI:** 10.3389/fimmu.2020.00613

**Published:** 2020-05-05

**Authors:** Mariapaola Marino, Umberto Basile, Gregorio Spagni, Cecilia Napodano, Raffaele Iorio, Francesca Gulli, Laura Todi, Carlo Provenzano, Emanuela Bartoccioni, Amelia Evoli

**Affiliations:** ^1^Istituto di Patologia Generale, Università Cattolica del Sacro Cuore, Rome, Italy; ^2^Fondazione Policlinico Universitario “A. Gemelli” IRCCS, Rome, Italy; ^3^Area Diagnostica di Laboratorio, Fondazione Policlinico Universitario “A. Gemelli” IRCCS, Rome, Italy; ^4^Istituto di Neurologia, Università Cattolica del Sacro Cuore, Rome, Italy; ^5^Dipartimento di Neuroscienze, Fondazione Policlinico Universitario “A. Gemelli” IRCCS, Rome, Italy; ^6^Istituto di Medicina Interna, Università Cattolica del Sacro Cuore, Rome, Italy; ^7^Area di Gastroenterologia e Oncologia Medica, Fondazione Policlinico Universitario “A. Gemelli” IRCCS, Rome, Italy; ^8^Dipartimento di Medicina di Laboratorio, Ospedale Madre Giuseppina Vannini, Rome, Italy

**Keywords:** rituximab, antibodies, MuSK, myasthenia gravis, IgG4, short-lived antibody-secreting cells, plasmablasts, plasma cells

## Abstract

The use of rituximab (RTX), an anti-CD20 monoclonal antibody (Ab), in refractory myasthenia gravis (MG) is associated with a better response in patients with Abs to the muscle-specific tyrosine kinase (MuSK) than in other MG subgroups. Anti-MuSK Abs are mostly IgG4 with proven pathogenicity and positive correlation with clinical severity. The rapid and sustained response to RTX may be related to MuSK Ab production by short-lived Ab-secreting cells derived from specific CD20^+^ B cells. Here, we investigated the long-term effects of RTX in nine refractory MuSK-MG patients with a follow-up ranging from 17 months to 13 years. In patients’ sera, we titrated MuSK-specific IgG (MuSK-IgG) and MuSK-IgG4, along with total IgG and IgG4 levels. Optimal response to RTX was defined as the achievement and maintenance of the status of minimal manifestations (MM)-or-better together with *a* ≥ 50% steroid reduction, withdrawal of immunosuppressants, and no need for plasma-exchange or intravenous immunoglobulin. After a course of RTX, eight patients improved, with optimal response in six, while only one patient did not respond. At baseline, MuSK-IgG and MuSK-IgG4 serum titers were positive in all patients, ranging from 2.15 to 49.5 nmol/L and from 0.33 to 46.2 nmol/L, respectively. MuSK Abs mostly consisted of IgG4 (range 63.80–98.86%). RTX administration was followed by a marked reduction of MuSK Abs at 2–7 months and at 12–30 months (*p* < 0.02 for MuSK-IgG and *p* < 0.01 for MuSK-IgG4). In patients with a longer follow-up, MuSK Ab titers remained suppressed, paralleling clinical response. In the patient who achieved long-term complete remission, MuSK-IgG4 was no longer detectable within 2 years, while MuSK-IgG remained positive at very low titers up to 10 years after RTX. In the patient who did not respond, MuSK-IgG and MuSK-IgG4 remained unchanged. In this patient series, total IgG and IgG4 transiently decreased (*p* < 0.05) at 2–7 months after RTX. The different trends of reduction between MuSK-IgG4 and total IgG4 after RTX support the view that short-lived Ab-secreting cells are the main producers of MuSK Abs. The ratio between short-lived Ab-secreting cells and long-lived plasma cells may influence the response to RTX, and B-cell severe depletion may reduce self-maintaining autoimmune reactivity.

## Introduction

Rituximab (RTX) is a chimeric mouse/human monoclonal antibody (Ab) directed against the B-lymphocyte membrane protein CD20. RTX acts through complement-mediated cytotoxicity, Ab-dependent cell-mediated cytotoxicity, and induction of apoptosis, leading to a profound depletion of circulating naïve and memory B cells (1). Since plasma cells do not express CD20, RTX administration does not directly affect immunoglobulin (Ig) levels (2). Originally licensed for the treatment of non-Hodgkin B-cell lymphoma, RTX has been used in a broad range of autoimmune diseases. Its therapeutic effect is conceivably related to both the Ab-dependent and Ab-independent (antigen presentation and cytokine production) roles of B lymphocytes in the immune system (3). RTX has proved particularly effective in the treatment of IgG4-related disease, a multi-organ fibro-inflammatory condition (4), as well as in IgG4-mediated autoimmune conditions (5).

IgG4 develops after prolonged antigen exposure and constitutes the least represented IgG subclass. IgG4 Abs may exert a protective role in allergy and are generally considered non-pathogenic, owing to their inability to activate complement and to cross-link identical antigens after Fab-arm exchange (6). More recently, the capacity of IgG4 Abs to directly interfere with the antigen function has been recognized and associated with their causative role in disease (7).

Myasthenia gravis (MG) is a rare disease of the neuromuscular junction caused by Abs against postsynaptic membrane proteins, such as the acetylcholine receptor (AChR), the muscle-specific tyrosine kinase (MuSK), or the low-density receptor-related protein 4 (LRP4). MG with Abs to MuSK (MuSK-MG) was one of the first diseases in which IgG4 pathogenicity was demonstrated (8) and currently represents a prototype of IgG4-mediated autoimmunity.

MuSK Abs are present in 5–7% of MG patients in association with a phenotype dominated by bulbar and neck weakness, are predominantly of the IgG4 subclass (9), and correlate with disease severity (10). When changes in clinical status and MuSK-specific IgG (MuSK-IgG) subclasses were investigated, only IgG4 levels were related to disease severity (11), and the IgG4 fraction, purified from patients’ serum, proved pathogenic *in vitro* (12, 13) and in passive transfer studies (8). MuSK Abs mostly bind the extracellular Ig1-like domain, which is crucial for MuSK–LRP4 interaction and AChR clustering (12). According to recent investigations, monovalent MuSK-IgG4, derived from Fab-arm exchange, are present *in vivo*, in patient serum (13). These bispecific Abs are chief players in the inhibition of agrin-induced MuSK activation, while monospecific MuSK Abs would rather have a protective effect by enhancing MuSK phosphorylation (14).

RTX-induced B-cell depletion was reported to be effective in patients with refractory MG, although with some variability in the degree of clinical response and reduction of pathogenic Abs (15–19). Data from clinical reports (15, 16, 19) and meta-analyses (20, 21) consistently showed a more pronounced and long-standing response in patients with MuSK Abs. In these cases, improvement in clinical status was paralleled by a prolonged reduction of MuSK-IgG4 (15).

The present study investigated the effect of RTX in patients with refractory MuSK-MG, together with MuSK-IgG and MuSK-IgG4 and total IgG and IgG4 serum concentrations. We found a correlation between variations in clinical status and changes in MuSK Ab titers. Treatment with RTX induced a marked reduction of MuSK-IgG4 while the serum levels of total IgG4 did not change significantly.

## Patients and Methods

### Patients

We retrospectively evaluated nine MuSK-MG patients treated with RTX at Policlinico Gemelli, Università Cattolica. All patients were diagnosed with refractory MG by meeting one of the following criteria: (1) presence of disabling weakness or MG relapses despite adequate treatment (prednisone plus immunosuppressants); (2) inability to reduce steroid dosage because of disease worsening on prednisone tapering, requiring repeated courses of plasma-exchange or intravenous immunoglobulin (IVIg) (at least three per year) and serious side effects from treatment (22).

In these subjects, maximum disease severity ranged from classes IIIb to V according to the Myasthenia Gravis Foundation of America (MGFA) classification (23); two patients had focal muscle atrophy; all were intolerant of pyridostigmine. All patients had received prednisone as initial treatment at a daily dose ≥1 mg/kg of body weight, followed by chronic administration every other day, when possible. Because of symptom relapse on prednisone tapering, immunosuppressive agents and emergency treatment (24), mostly with plasma-exchange, were required in all cases. Seven patients had tried two or more immunosuppressants (azathioprine, AZA; mycophenolate mofetil, MMF; and cyclosporine A, CyA); two patients had received AZA for at least 1 year with no response, when we decided to administer RTX on account of disabling weakness, short-term benefit from plasma-exchange, and serious side effects from steroids (diabetes and osteoporosis in both cases). One of these patients (#5 in Table 1) had received a course of RTX 6 years previously in another hospital, with reported benefit; from 1994 to 2000, before MuSK-MG onset, she had suffered from relapsing thrombotic thrombocytopenic purpura treated with high-dose steroids, IVIg, and splenectomy.

**TABLE 1:** Rituximab in refractory MuSK-MG patients.

**Pt # gender**	**Age at onset (years)**	**Age at RTX**	**Max MGFA**	**MGFA at RTX**	**Therapy at RTX (daily dosage)**	**Post-RTX best PIS**	**Response**	**Response duration (months)**	**RTX cycles**	**Follow-up after RTX (months)**
#1. F	61	64	V	IIIb	P (25 mg), AZA (150 mg), PE	MM	optimal	30, ongoing	1	30
#2. F	46	62	V	IIb	P (25 mg), MMF (1.5 g), PE	CSR	optimal	42	2*	46
#3. F	30	44	V	IIIb	P (20 mg), MMF (2 g), PE	MM	optimal	28	1	31
#4. M	38	47	IIIb	IIIb	P (30 mg), MMF (2 g), PE	U	no	-	2**	53
#5. F	23	38	V	IIIb	P (37.5 mg), MMF (2 g)	I	partial	24	1	48
#6. F	29	40	V	IIb	P (40 mg), CyA (175 mg)	PR	optimal	71, ongoing	1	71
#7. F	42	48	IVb	IIIb	P (30 mg), MMF (2 g), PE	CSR	optimal	144, ongoing	1	144
#8. F	17	38	IIIb	IIIb	P (30 mg), MMF (2 g)	MM	optimal	20, ongoing	1	20
#9 F	69	73	V	IIIb	P (20 mg), AZA (150 mg)	I	partial	23, ongoing	1	23

MGFA: Myasthenia Gravis Foundation America clinical status; P: prednisone; AZA: azathioprine; PE: plasma-exchange; MMF: mycophenolate mofetil; CyA: cyclosporine A.PIS: post-intervention status; CSR: complete stable remission; PR: pharmacological remission; MM: minimal manifestations; I: improved; U: unchanged. *Pt #2 received a second RTX cycle on Sep 2019. **Pt #4 received a second RTX cycle on Oct 2018 with improvement. Follow-up after RTX = at last visit.

RTX was administered according to the non-Hodgkin lymphoma regimen adopted in our institution. A RTX course consisted of an infusion of 375 mg/m^2^ once a week for four consecutive weeks, plus a single dose of 375 mg/m^2^ after 3 months. Infusion was preceded by intravenous methylprednisolone and oral antihistamine medication to minimize acute reactions.

Before treatment, patients were screened for active infections including viral hepatitis (B and C) and tuberculosis (25) and for significant arrhythmias and ischemic heart disease (26).

We classified response to RTX considering changes in both clinical status and treatment. Optimal response was defined by the achievement and maintenance of the status of minimal manifestations (MM) or better (23), together with a ≥ 50% reduction of steroid dose, withdrawal of immunosuppressants, no need for plasma-exchange, or IVIg. Patients had a partial response when, although improved, they failed to achieve the status of MM-or-better or when prednisone reduction was<50% of pretreatment dosage, with or without immunosuppressant withdrawal, no need for plasma-exchange, or IVIg. No response corresponded to nochange in clinical status together with minimal or no variations in the required treatment.

Clinical changes were assessed as post-intervention status (PIS) (23).

### Laboratory Assays

Fifty-two serum samples from the nine patients included in the study (four to seven samples per patient) were collected before and after RTX treatment. Samples were stored at −20°C until the analysis was performed.

MuSK-IgG titration was performed using human recombinant ^125^I-labeled MuSK (RSR Limited, Cardiff, United Kingdom; cutoff ≥ 0.05 nmol/L), according to the manufacturer’s instruction, with minor modifications. In brief, 50 μl of a 10X serum dilution (in assay buffer) was incubated overnightwith 50 μlof ^125^I-MuSK. The antigen/Ab complexes were precipitated by the addition of 50 μl of goat anti-human IgG and separated by centrifugation. Serum samples with saturating titers were further analyzed at different dilutions with normal human serum (NHS, from 10X to100X) (10).

MuSK-IgG4 Abs were determined as described by Tsiamalos and coworkers with minor modifications (27). Briefly, 20 μl of ^125^I-MuSK solution [from the aforementioned radioimmunoassay (RIA)] was incubated with 0.2 μl of patients’ serum for 8–12 h at 4°C (brought to a final volume of 20 μl with assay buffer). Serum samples were previously diluted with NHS to maintain the antigen/Ab binding in the linear zone of the slope. After adding5 μl of sheep anti-human IgG4 (Binding Site, Birmingham, United Kingdom), samples were incubated overnight at 4°C. Then, 15 μl of anti-sheep IgG anti-serum (Binding Site, Birmingham, United Kingdom) was added for 4 h at room temperature to precipitate MuSK-IgG4Abs complexed to ^125^I-MuSK. After final washes, the radioactivity was measured in a gammacounter. Results were expressed as ^125^I-MuSK nanomoles per liter. All samples from the same patient were titrated in the same batch to limit inter-assay variability.

Total IgG and IgG4 serum levels were measured by turbidimetry (human IgG and IgG subclass liquid reagent kits, The Binding Site) on the Optilite instrument according to the manufacturer’s recommendations. Normal range is 8–18 g/L for total IgG and 0.04–0.86 g/L for the IgG4 subclass. Serological analysis was performed blind of clinical data.

B lymphocytes in peripheral blood were evaluated as routine assessment in patients treated with RTX, by standard flowcytometry in our institution’s central laboratories. CD19^+^ cell normal values ranged from 100 to 400 cells/mm^3^ (7–16% of lymphocyte count).

### Statistical Analysis

For the statistical analysis, we converted actual serological titers into percentage values considering the baseline levels as 100%. We employed one-way ANOVA among three groups (baseline *vs* 2–7 months *vs* 12–30 months); Student’s*t* test was performed between two groups. A *p*-value < 0.05 was considered significant (Supplementary Table).

### Ethical Consideration

The ethic committee of our institution approved the study. All patients gave written informed consent to off-label treatment with RTX and to the use of their clinical and serological data in this study.

## Results

### Clinical Response

The study population included nine patients (eight females) with refractory MuSK-MG, treated with RTX between July 2006 and October 2018. The patients’ demographics, baseline and post-treatment clinical data, and follow-up duration are shown in Table 1.

Age at infusion ranged 38–73 years (mean 50.4 ± 12.8). The interval between disease onset and RTX administration varied between 3 and 21 years (mean 11 ± 6.04). At the time of treatment, MG severity ranged from IIb to IIIb according tothe MGFA classification (23), and all patients were taking prednisone plus one immunosuppressant; five of nine patients were on maintenance treatment with plasma-exchange.

Optimal response to RTX was recorded in six of nine patients (66.6%). Prednisone was tapered off in two patients (#2 and #7) and was reduced by 75% to 87.5% of the pretreatment dosage (actual doses of 5–10 mg every other day) in the others. After a single course of RTX, patient #7 achieved electromyography (EMG)-confirmed complete stable remission (CSR), persisting at the last follow-up, 13 years after RTX administration and 5 years from immunosuppression withdrawal. In the other patients, optimal response to RTX (PIS ranging from CSR to MM) lasted 28–42 months in patients #2 and #3, and is still ongoing 20 to 71 months after RTX, in patients #1, #6, and #8 (see Table 1). A second course of RTX has just been completed in patient #2 and has been planned for patient #3.

Two patients showed a partial response since they did not achieve MM status, although prednisone and immunosuppressive therapy were reduced by 50%. In patient #9 who had developed a myopathic face, weakness of facial muscles remained unchanged, while dysarthria and neck weakness markedly improved. Patient #4, suffering from tongue wasting and severe bulbar weakness, did not improve after a first cycle of RTX, while he responded to a second course with improvement of dysarthria and partial reversal of tongue atrophy. RTX was well tolerated with no infusion reactions or long-term side effects.

### MuSK Abs and MuSK-IgG Profile After Treatment

**FIGURE 1:**
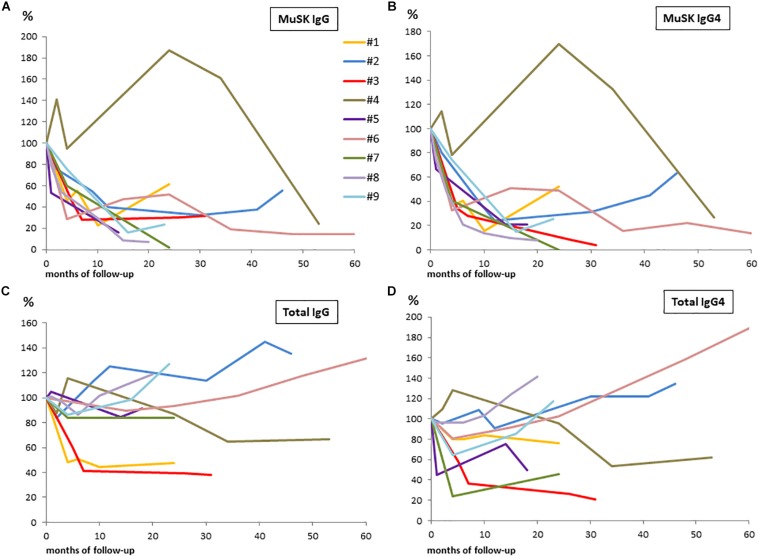
Changes from baseline to 60 months after RTX in patients serum samples are shown in panels: **(A)** for MuSK-IgG; **(B)** for MuSK-IgG4; **(C)** for total IgG; and **(D)** for total IgG4. We converted actual serological titers into percentage values considering the baseline levels as 100%.

The results of serological assays, together with PIS and changes in B-cell count, are reported in Table 2. Changes from baseline to 60 months after RTX are shown in Figure 1 (panels: A for MuSK IgG, B for MuSK IgG4, C for total IgG, D for total IgG4). At baseline, MuSK-IgG and MuSK-IgG4 serum titers were positive in all patients, ranging from 2.15 to 49.5 nmol/L and from 0.33 to 46.2 nmol/L, respectively. MuSK Abs mostly consisted of IgG4 (range 63.80–98.86%) in all patients but one (#5, not included in the range), in whom the MuSK-IgG4/MuSK-IgG ratio was 15.34%.

**TABLE 2:** Serological and clinical profiles in MuSK-MG patients after treatment with rituximab.

**Pt**	**Months from RTX**	**anti-MuSK IgG (nmol/L)**	**anti-MuSK IgG4 (nmol/L)**	**IgG (g/L)**	**IgG4 (g/L)**	**MGFA class/PIS**	**CD19+ n°. x mm^3^ (% lymphocytes)**
**1**	0	11.85	11.7	14.39	0.25	IIIb	292 (10.6%)
	4	5.4	4.25	6.95	0.20	Improved	13 (0.9%)
	6	6.6	4.71	7.31	0.20	Improved	36 (1.6%)
	10	2.7	1.84	6.39	0.20	MM	32 (0.1%)
	24	7.3	6.1	6.86	0.19	MM	21 (1.5%)

**2**	0	32.4	25.3	8.56	0.23	IIb	200 (9.4%)
	2	24.5	20.8	7.20	0.22	Improved	n.d.
	6	17.7	10.1	9.74	0.25	MM	9 (0.4%)
	12	13	6.1	10.71	0.21	PR	18 (0.8%)
	30	10.6	8	9.76	0.28	CSR	352 (15.8%)
	41	12.1	11.4	12.39	0.28	CSR	341 (18.1%
	46	17.9	16	11.56	0.31	IIb*	n.d.

**3**	0	7.57	7.17	22.04	0.19	IIIb	118 (11%)
	5	3.44	2.44	13.32	0.11	improved	16 (0.5%)
	7	2.14	2.03	9.09	0.07	MM	9 (0.3%)
	26	2.3	0.6	8.68	0.05	MM	47 (2.5%)
	31	2.4	0.3	8.43	0.04	IIb	181 (6.4%)

**4**	0	10.57	9.79	8.6	0.21	IIIb	138 (15%)
	2	14.9	11.2	7.55	0.23	Unchanged	15 (1%)
	4	10	7.7	9.93	0.27	Unchanged	n.d.
	24	19.8	16.6	7.51	0.20	Unchanged	n.d
	34	17	13	5.55	0.11	Unchanged	n.d
	53	2.6	2.6	5.73	0.13	Improved**	n.d

**5**	0	2.15	0.33	6.33	0.20	IIIb	193 (4%)
	1	1.15	0.22	6.65	0.09	Unchanged	9 (0.2%)
	14	0.34	0.07	5.33	0.15	Improved	11 (0.4%)
	18	n.d.	0.07	5.77	0.10	Improved	11 (0.3%)

**6**	0	18.07	11.53	7.30	0.36	IIb	100 (9.1%)
	4	5.2	3.8	7.11	0.29	PR	2 (0.1%)
	15	8.49	5.84	6.54	0.33	PR	0
	24	9.4	5.65	6.83	0.37	PR	29 (2.6%)
	36	3.38	1.84	7.42	0.47	PR	n.d.
	48	2.6	2.57	8.56	0.57	MM	100 (9.6%)
	60	2.7	1.6	9.61	0.68	MM	142 (10.5%)
	71	2.7	2.4	10.36	0.81	MM	n.d.
**7**	0	12.24	12.1	14.59	1.07	IIIb	n.d.
	4	7.34	4.87	12.22	0.26	Improved	n.d.
	24	0.21	0.01	12.23	0.49	MM	n.d.
	80	0.18	0.01	11.78	1.07	CSR	n.d.
	120	0.21	0.01	11.96	1.15	CSR	n.d.
	132	0	0	12.23	1.19	CSR	n.d.

**8**	0	13	11.4	11.46	0.58	IIIb	298 (12.3%)
	1	10.3	8.5	11.58	0.56	Unchanged	0
	3	7	5.5	11.06	0.56	MM	0
	6	5.7	2.4	9.53	0.56	MM	7 (0.67%)
	10	3.8	1.6	11.64	0.60	MM	N/D
	15	1.1	1.1	12.70	0.72	MM	N/D
	20	0.94	0.9	13.59	0.82	MM	N/D

**9**	0	49.5	46.2	10.99	0.34	IIIb	107 (11.8%)
	4	37.3	34.3	9.47	0.22	Improved	0
	16	8.1	7	10.81	0.29	Improved	N/D
	23	11.8	11.8	13.96	0.40	Improved	157 (17%)

Pt: patient; MGFA: Myasthenia Gravis Foundation America clinical status; PIS: post interventional status; MM: minimal manifestations; PR: pharmacological remission, CSR: complete stable remission. Normal range for IgG: 8-18 g/L. Normal range for IgG4 subclass: 0.04–0.86 g/L. *Pt #2 received a second RTX cycle on Sep, 2019. **Pt #4 received a second RTX cycle on Oct, 2018 with improvement.

**FIGURE 2:**
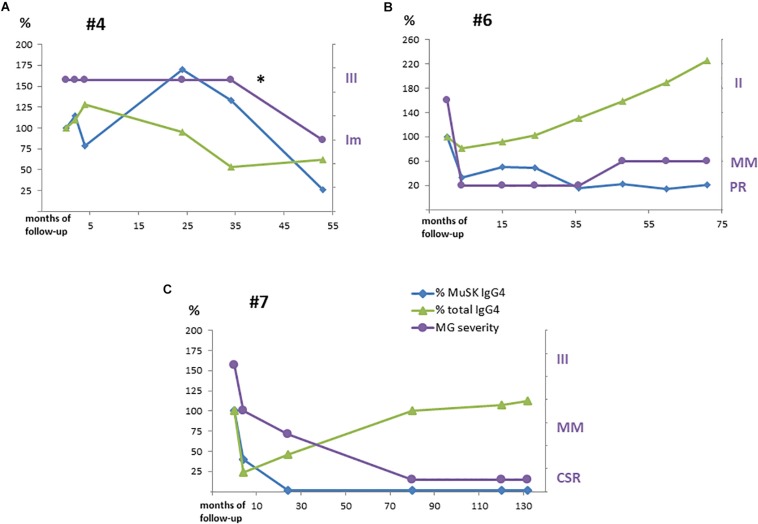
MuSK-IgG4 and total IgG4 subclass levels after RTX treatment along with variations in clinical severity are shown in panels: **(A)** for #4, **(B)** for #6, and **(C)** for #7. Serological titers were converted into percentage values considering the baseline levels as 100%. MG severity was referred to as MGFA/PIS as reported in Table 2. *Pt #4 received a second RTX with improvement (Im).

MuSK-IgG and MuSK-IgG4 titers did not change after a course of RTX in the only patient (#4) who did not respond (Figure 2A), while, in the other patients, they decreased to a different extent, paralleling clinical improvement (Figure 2B). In patient #7, who achieved CSR, MuSK-IgG and MuSK-IgG4 were greatly reduced and became negative after RTX. However, while MuSK-IgG was still detectable at very low levels up to 10 years from treatment, MuSK-IgG4 was undetectable 2 years after RTX and remained negative in subsequent assays (Figure 2C).

For statistical analysis, we compared MuSK-IgG and MuSK-IgG4 baseline levels with those at 2–7 months and at 12–30 months after RTX in eight patients (samples from patient #5 were not available). We found a significant reduction at both time points (*p* < 0.02 for MuSK-IgG and *p* < 0.01 for MuSK-IgG4, by ANOVA), while the MuSK-IgG4/MuSK-IgG ratio was reduced significantly only at 2–7 months after RTX (*p* < 0.05, by Student’s *t* test).

In patients who experienced prolonged benefit from RTX, MuSK Ab titers remained suppressed, regardless of B-cell count normalization (see Table 2).

At baseline, in two of nine patients, total IgG serum levels were lower than those in normal controls. The relative proportions of the IgG subclasses were within the normal range in eight of nine patients (data not shown). Patient #7 had a higher IgG4/IgG ratio (7.3%) that persisted (after a temporary decrease in the first 2 years after RTX) in the long-term follow-up, when MuSK-IgG4 was no longer detectable.

Total IgG and IgG4 did not significantly change at 2–7 and 12–30 months after RTX when compared to the baseline levels (*p* = 0.15 for total IgG; *p* = 0.17 for IgG4, by ANOVA). However, we found a significant reduction when comparing baseline levels with those at 2–7 months (*p* < 0.05 for total IgG and IgG4, by Student’s *t* test); afterward, total IgG and IgG4 returned to pretreatment levels. The total IgG4/IgG ratio was unchanged at both time points.

## Discussion

In line with previous reports (15, 18–21, 28, 29), our data confirm that, in patients with MuSK-MG, RTX is safe and induces long-term benefit associated with a strong steroid- and immunosuppressant-sparing effect. As MuSK-MG is often a life-threatening disease with a high proportion of patients refractory to conventional therapy (30, 31), RTX has been proposed as an early therapeutic option in patients unresponsive to first-line immunosuppression (32).

The rapid and sustained response to RTX suggests that MuSK Abs are mostly produced by short-lived Ab-secreting cells (33, 34), a cell pool that needs to be constantly refilled from the B-cell compartment (35). In contrast, bone marrow long-lived plasma cells, which are crucial for maintaining serum IgG concentration, are known to be scarcely affected by RTX (3). Recent studies, in MuSK-MG patients, showed that auto-Ab-expressing CD27^+^ B cells are present in the peripheral blood during disease relapses after RTX and that circulating CD20^–^CD27^high^ CD38^+^plasmablasts contribute to MuSK Ab production (36). In this model, supported by consistent findings in other IgG4-mediated diseases (37, 38), the therapeutic effect of RTX would be mostly related to depletion of plasmablast precursors (36, 39).

A comparison of our results with other studies is limited by differences in MG severity and therapy at baseline, follow-up duration, and treatment protocol. Our decision to repeat RTX was entirely clinical, based on reappearance of disabling symptoms. The majority of our patients received a single course of RTX that granted most of them a status of MM-or-better, including one case of long-standing CSR with MuSK Ab negativization. Clinical response lasted 24–42 months in patients who relapsed after RTX. It is still ongoing in the other patients after a clinical observation of up to 71 months. These findings confirm earlier reports of a long-standing effect of RTX in MuSK-MG (15, 18, 19, 29, 40). On the other hand, three (33.3%) of our patients had a less satisfactory outcome than usually reported in this MG subtype. It is worth mentioning that two of these subjects had developed focal muscle atrophy. As, in this series, a second RTX course brought about a clear benefit in a previously unresponsive patient, timely re-treatment may be required in these cases.

Our results confirm the correlation between MuSK Abs and clinical status (11, 15), as in all RTX-responsive patients, MuSK-IgG and MuSK-IgG4 serum titers were significantly reduced and, in most of these cases, remained suppressed regardless of B-cell count normalization(patients #2–3–6–9 in Table 2).

In our population, IgG4 was the main MuSK Ab isotype in all our patients but one, in whom IgG4 accounted for 15.3% of MuSK Ab titer. This finding might be related to the patient’s medical history (splenectomy for thrombotic thrombocytopenic purpura and previous treatment with RTX). In patient #2, an increase in serum MuSK Abs, and above all, in MuSK-IgG4 heralded clinical deterioration. Conversely, in patient #7, who achieved long-standing CSR, MuSK-IgG4 was no longer detectable several years before MuSK-IgG negativization. Overall, these data agree with the view that MuSK-IgG4 is pathogenic and is mostly produced by short-lived Ab-secreting cells.

There is no established protocol for RTX administration in MG, although the non-Hodgkin lymphoma regimen has been the most common induction treatment (21). Recently, in a multicenter study investigating the response to different RTX dosages, the protocol of 375 mg/m^2^/week for 4 weeks plus an infusion monthly for the next 2 months, which is similar to that adopted in our study, was associated with the lowest relapse rate in MuSK-MG (41). RTX cycles were repeated based on clinical relapses in some studies (15, 16, 29) or per protocol in others (17, 19, 28, 42), with an overall positive correlation between number of treatment courses and clinical outcome (18). Considering MuSK-MG pathogenesis, reemerging CD27^+^ B cells and plasmablasts in peripheral blood can serve as a valuable reference for timing re-treatment. As an increase in MuSK-IgG (and MuSK-IgG4) can herald a clinical relapse, changes in Ab titers may be used to monitor clinical response.

It is well known that RTX is highly effective in IgG4-associated diseases and can deplete short-lived Ab-secreting cells without affecting long-lived plasma cells [for a review, see (43)]. The effect of RTX on the longitudinal levels of total serum IgG4, in MuSK-MG, has not been investigated. In our population, RTX administration did not affect total IgG4 to a greater extent than other IgG subclasses, as total IgG and IgG4 serum levels both decreased in the first months after treatment and then returned to normal. From our data, total IgG and IgG4 appear to be mostly produced by long-lived plasma cells, even though short-lived Ab-secreting cells, may contribute. On the other hand, the long-lasting decline of MuSK-Abs, particularly of IgG4 isotype, supports the view that short-lived Ab-secreting cells are the main producers of MuSK Abs.

Overall, our data show that the therapeutic effects of RTX can persist for several years after treatment, suggesting that, by depleting autoreactive B-cell clones, RTX may significantly disrupt the immunopathogenic circuit responsible for disease maintenance. It is well known that B-cell activity depends on T–B lymphocyte cross talk and cooperation. Future studies should investigate how RTX affects such an interaction, particularly regarding specific T- and B-cell repertoires.

## Data Availability Statement

The datasets generated for this study are available on request to the corresponding author.

## Ethics Statement

The studies involving human participants were reviewed and approved by Università Cattolica del Sacro Cuore, Fondazione Policlinico Universitario “A. Gemelli” – I.R.C.C.S. The patients/participants provided their written informed consent to participate in this study. Written informed consent was obtained from the individual(s) for the publication of any potentially identifiable images or data included in this article.

## Author Contributions

MM, EB, and AE wrote the manuscript. MM, UB, and EB designed the experiments. GS, RI, and AE collected the serum samples and clinical data. UB, CN, FG, LT, and CP performed the experiments. EB and AE analyzed the data.

## Conflict of Interest

The authors declare that the research was conducted in the absence of any commercial or financial relationships that could be construed as a potential conflict of interest.
