# Supplementary material for: Long-Lasting Rituximab-Induced Reduction of Specific—But Not Total—IgG4 in MuSK-Positive Myasthenia Gravis
Source: Front Immunol. 2020 May 5;11:613. doi: 10.3389/fimmu.2020.00613 (PMC7214629; doi:10.3389/fimmu.2020.00613)
Supplement: Supplementary file 1 [file Table_1.DOCX]

**Supplemental Table: statistical analysis.**

| **Patients: 8/9** | **baseline** | **2-7 months**  **(mean ± SD)** | **12-30 months (mean ± SD)** | ***p*** | **test** |
| --- | --- | --- | --- | --- | --- |
| anti-MuSK IgG (%) | 100 | 58.83 ± 22.00 | 48.89 ± 59.37 | 0.018477 | **ANOVA** between groups |
| anti-MuSK IgG4 (%) | 100 | 51.65 ± 22.62 | 42.58 ± 54.77 | 0.005295 | **ANOVA** between groups |
| total IgG (%) | 100 | 83.34 ± 22.64 | 86.4 ± 29.27 | n.s.* | **ANOVA** between groups |
| total IgG4 (%) | 100 | 77.2 ± 32.10 | 86.22 ± 37.44 | n.s.** | **ANOVA** between groups |

n.s.: not significant

*****Student’s T: baseline *vs* 2-7: 0.037929

******Student’s T: baseline *vs* 2-7: 0.042258
